# The *Saccharomyces* Genome Database Variant Viewer

**DOI:** 10.1093/nar/gkv1250

**Published:** 2015-11-17

**Authors:** Travis K. Sheppard, Benjamin C. Hitz, Stacia R. Engel, Giltae Song, Rama Balakrishnan, Gail Binkley, Maria C. Costanzo, Kyla S. Dalusag, Janos Demeter, Sage T. Hellerstedt, Kalpana Karra, Robert S. Nash, Kelley M. Paskov, Marek S. Skrzypek, Shuai Weng, Edith D. Wong, J. Michael Cherry

**Affiliations:** Department of Genetics, Stanford University School of Medicine, Stanford, CA 94305, USA

## Abstract

The *Saccharomyces* Genome Database (SGD; http://www.yeastgenome.org) is the authoritative community resource for the *Saccharomyces cerevisiae* reference genome sequence and its annotation. In recent years, we have moved toward increased representation of sequence variation and allelic differences within *S. cerevisiae*. The publication of numerous additional genomes has motivated the creation of new tools for their annotation and analysis. Here we present the Variant Viewer: a dynamic open-source web application for the visualization of genomic and proteomic differences. Multiple sequence alignments have been constructed across high quality genome sequences from 11 different *S. cerevisiae* strains and stored in the SGD. The alignments and summaries are encoded in JSON and used to create a two-tiered dynamic view of the budding yeast pan-genome, available at http://www.yeastgenome.org/variant-viewer.

## INTRODUCTION

The *Saccharomyces* Genome Database (SGD; http://www.yeastgenome.org) is a community and bioinformatic resource distributing published facts and accumulated knowledge regarding yeast chromosomes, genes, gene products and their associated functions and interactions. The first completed eukaryotic genome sequence was that of *Saccharomyces cerevisiae* strain S288C, and was released in 1996 (1). Since the completion of the resequencing and updated annotation of the S288C genome, released in 2014 (2), we have turned our efforts toward the sequencing and annotation of additional genomes from different strain backgrounds. Despite being less genetically diverse than other *Saccharomyces* species, such as *S. paradoxus*, *S. cerevisiae* is extremely varied with respect to its allele and gene complements (3). Researchers continue to explore the ways in which these differences contribute to metabolic and phenotypic variation. The advent of high-throughput sequencing has allowed researchers to calibrate experiments based on the true genomic sequence of a particular strain. Recently we have been collecting publicly deposited strain sequences, and have commissioned the sequencing of 11 high profile yeast strains for inclusion and annotation (Table 1; (4)). These genomes were selected based on their extensive histories of use, and the availability of substantial amounts of published experimental results. To increase utility of the new genome sequences, SGD will provide comprehensive annotation and comparative analyses, correlating changes with variations in phenotypes and protein function. As part of our initial efforts, we have developed a new Variant Viewer to explore and highlight differences in DNA and protein sequences. While other tools, such as the NCBI Variation Viewer (5) and Yeast Resource Center Snip Viz (6), exist to visualize genetic variation, the SGD Variant Viewer is distinguished by its ability to simultaneously visualize high-level differences of many genes, as well as sequence changes in individual features. Additionally, it can be repurposed for use on other websites. The Variant Viewer has been made available at GitHub and registered with BioJS (7) along with sample integration code to promote open sharing and support scientific discovery.

**Table 1.** Genomes included in the initial release of the Variant Viewer

Strain	SGD strain page URL	Provenance	Accession
CEN.PK2-1Ca	http://www.yeastgenome.org/strain/CENPK/overview	Laboratory strain	JRIV00000000
D273-10B	http://www.yeastgenome.org/strain/D273-10B/overview	Laboratory strain	JRIY00000000
FL100	http://www.yeastgenome.org/strain/FL100/overview	Laboratory strain	JRIT00000000
JK9-3d	http://www.yeastgenome.org/strain/JK9-3d/overview	Laboratory strain	JRIZ00000000
RM11-1A	http://www.yeastgenome.org/strain/RM11-1a/overview	Derivative of California vineyard isolate	JRIP00000000
Σ1278b-10569-6B	http://www.yeastgenome.org/strain/Sigma1278b/overview	Laboratory strain	JRIQ00000000
SEY6210	http://www.yeastgenome.org/strain/SEY6210/overview	Laboratory strain	JRIW00000000
SK1	http://www.yeastgenome.org/strain/SK1/overview	Laboratory strain	JRIH00000000
W303	http://www.yeastgenome.org/strain/W303/overview	Laboratory strain	JRIU00000000
X2180-1A	http://www.yeastgenome.org/strain/X2180-1A/overview	S288C-derived laboratory strain	JRIX00000000
Y55	http://www.yeastgenome.org/strain/Y55/overview	Laboratory strain	JRIF00000000

All genome sequences were determined using the AGAPE pipeline (4).

## PREPARATION OF VARIANT DATA

Multiple sequence alignments for open reading frames (ORFs) and translation products from 11 strains of *S. cerevisiae* (Table 1) and the S288C reference genome were generated using Clustal Omega (8). Variant sites within the aligned sequences were identified, noting both the position and the type of variation (single nucleotide polymorphism (SNP), insertion or deletion), as well as the type of SNP (synonymous, non-synonymous, or intronic) when appropriate.

Similarity scores were generated for each ORF sequence in each strain relative to S288C as the proportion of matching residues. For each aligned nucleotide or amino acid sequence, the total number of matches from corresponding positions to the S288C reference are recorded and divided by the total length of the full alignment. Therefore, if the sequence is identical to the reference, the score for that strain will be exactly one. In this scheme, a score of zero would indicate no matching residues. If 90% of the sequence matches, then a score of 0.9 is assigned. If no comparison can be made because the gene is absent in a particular strain, or because the sequence data are not currently available, then the score is null for that cell and is listed as ‘not available’ (N/A). Currently, the interface does not indicate which of these cells represent sequences that are actually missing from those for which the data are not yet available. Future iterations of the interface will make this distinction, as the sequences and their automated annotation obtained from the AGAPE output are actively undergoing manual curation to resolve these issues. To this end, the Variant Viewer has proven itself to be an extremely valuable tool for the identification of potentially problematic sequences and prioritization of manual sequence curation activities.

The amount of variation in the full set of all genes identified within a genome in *S. cerevisiae*, the ‘pan genome’, can be represented by two matrices (one each for DNA and protein), each composed of 11 columns (one for each strain) and over 6500 rows (one for each ORF). Each cell of the matrix represents the amount of variation for a particular ORF in a specific strain, relative to the S288C reference.

The full complement of variants in each ORF (SNP sequence) is used to generate a dendrogram that represents the clustering of the individual sequences. The SNP sequence is generated by parsing through the variant data generated from the alignment and combining the SNPs from each aligned sequence. The result of this step is a sequence for each strain with a length equal to the total number of SNPs in the ORF. From these sequences, a JavaScript implementation of hierarchical clustering is used to calculate and draw a dendrogram without the cost of comparing identical segments of sequence.

**Figure 1.**
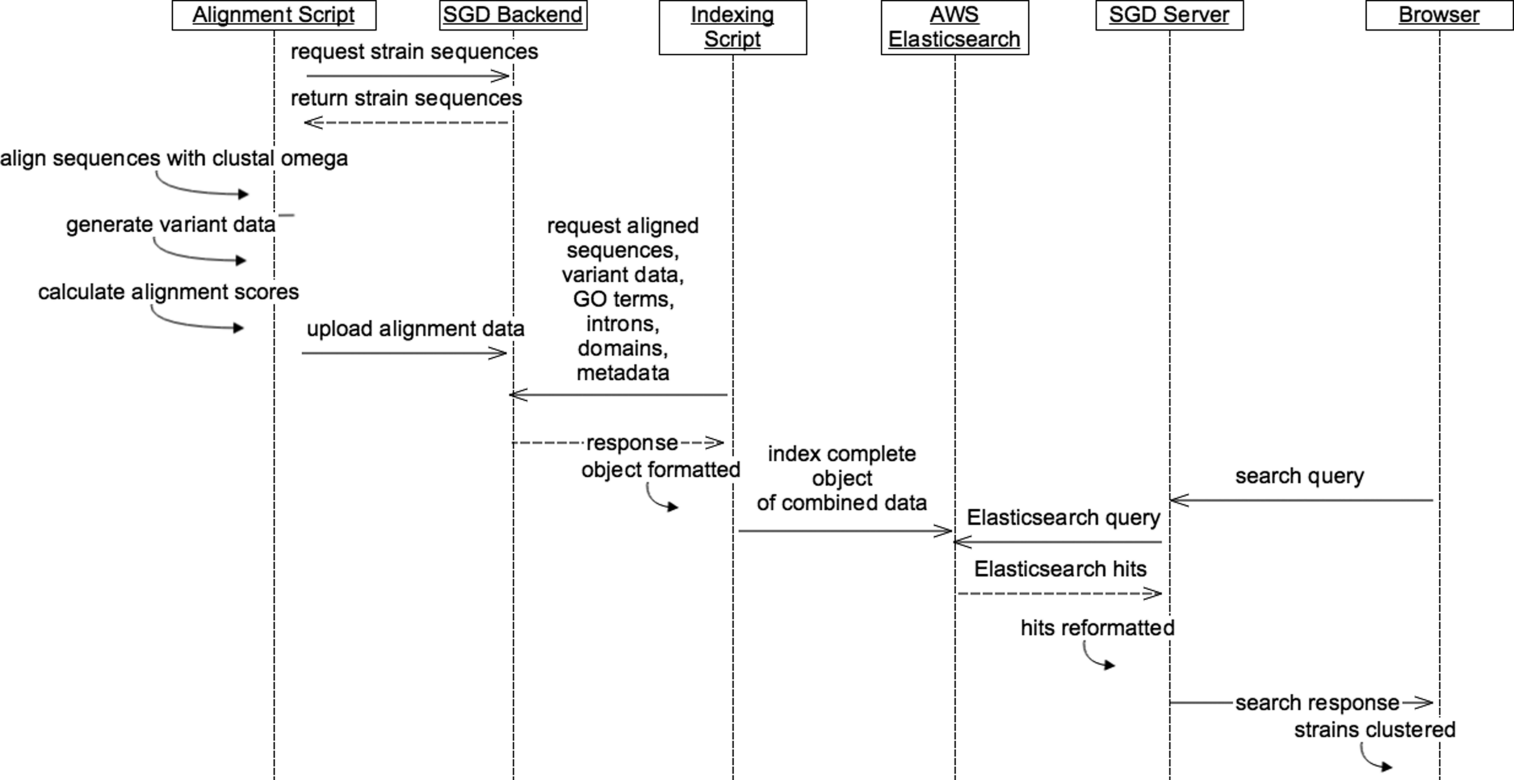
Pipeline of Variant Viewer data. The pipeline of variant data begins by fetching sequences for the reference strain and 11 alternative strains. The sequences for each feature are aligned using Clustal Omega (7) and stored in SGD's database. The alignment data is then combined with GO annotations, protein domains, introns for the reference genome and location in the reference genome to a JSON object that is indexed into Elasticsearch. When a user visits the site, the SGD server responds with the same JSON object, which the browser can use to render the interface.

The complete pipeline of data for the Variant Viewer starts with sequences from the SGD database and ultimately renders graphics in the browser with JavaScript (Figure 1). Sequences are first fetched from the SGD application program interface (API), then processed using Clustal Omega (7) to produce alignments. From the aligned sequences, an array of variant data is produced for both DNA and protein sequences. Each entry in these arrays notes the position of the variant relative to the aligned sequence, the variant type and the SNP type (if the variant is a SNP). These alignment data are stored in the database. The alignment data are then retrieved, along with protein domains, introns in the reference genomes, as well as Gene Ontology (GO) annotations, to produce a JSON object that can be indexed into an Elasticsearch (https://www.elastic.co/products/elasticsearch) instance. A visit to the Variant Viewer generates a request for data from an SGD server, that responds with the aforementioned JSON object of combined data.

## USER INTERFACE

**Figure 2.**
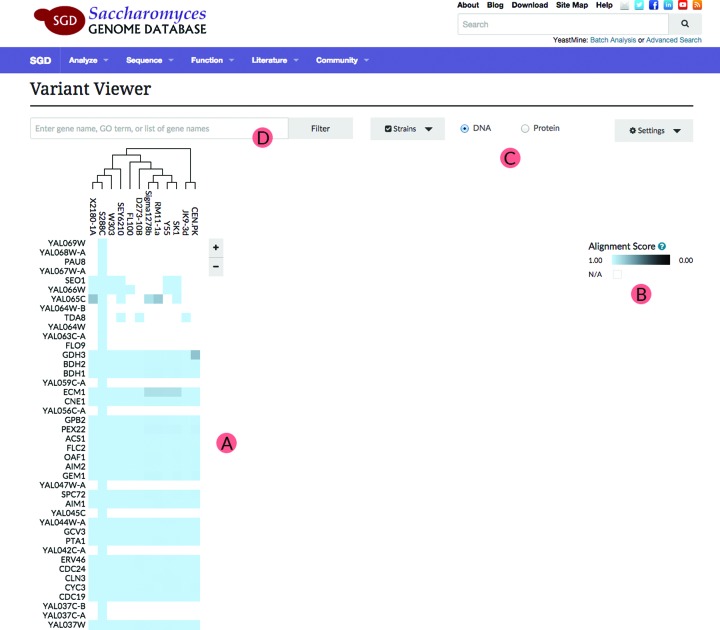
Matrix visualization of alignments. (**A**) A matrix is provided showing the available strains with a dendrogram illustrating their sequence relationships. The rows represent the ORFs and their variation relative to the reference. The table allows users to scroll through visible features. (**B**) The matrix is colored with a scale that corresponds to their alignment to S288C (**C**) User is allowed to toggle between DNA or protein variation view. (**D**) A search bar allows users to limit the features that appear in the display.

After navigating to SGD's Variant Viewer (http://yeastgenome.org/variant-viewer), the initial state of the application is displayed: a matrix of shaded nodes, with strain names along the x-axis and features along the y-axis (Figure 2). In the initial state, all available features are listed, and can be viewed by scrolling down the page. By default, scrolling moves along Chromosome I, and continues to Chromosome II and onward. The color value assigned to each square of the matrix is based on level of variation of a particular ORF in the corresponding strain, relative to the reference sequence. In this initial release, the reference is always S288C. Darker shades indicate more variation, and lighter shades indicate more conservation, with the lightest blue corresponding to 100% identity with the reference genome. White squares represent the absence of available data, either because the ORF is absent in a particular strain, or the necessary sequence data are not currently available. From this initial state, the interface is highly dynamic and interactive (Figure 2).

The interface provides users the option to filter data or focus on different data points. A radio selector along the top of the page allows toggling between DNA and protein modes (Figure 2C). These modes modify the color matrix according to the variation in the DNA or protein sequences of the visible features. A ‘Settings’ dropdown menu gives users control of how the features are sorted. Additionally, a search bar allows users to limit the features that appear in the display (Figure 2D). Users can search for features by chromosome, gene name, text in their descriptions or GO annotations. For example, a search term such as ‘kinase’ or ‘chromosome II’ displays only those genes relevant to that search term. The ‘Strain Selector’ dropdown provides a checkbox of available genomes, and changes which strains are displayed in the interface. Above the matrix, a dendrogram represents the degree of similarity of the selected ORFs in the currently selected strains. The dendrogram is dynamically redrawn whenever the features are filtered by searching or changing the selection of strains.

From the set of visible features, the previously described SNP sequences are combined to generate a SNP sequence for each strain across the resulting features. From the strains’ SNP sequences, a hierarchical clustering algorithm can generate a JSON representation of a tree. An open-source JavaScript implementation of bottom-up agglomerative hierarchical clustering is used for this task (https://github.com/harthur/). This library takes two inputs: an array of data and a function to compare any set of two elements from the array and return a floating point representation of the distance. The input array contains a JavaScript object for each strain. Each strain element has the combined SNP sequence for the visible set of filtered features. The comparison function takes any pair of strains, and compares their SNP sequences. For each residue in the sequence, it looks at the same position in the corresponding sequence of the comparison strain. It counts the total number of differing sequence characters, and then divides by the number of nucleotides in the selected features in the reference genome. If the sequences are identical, then the distance will be 0. In a SNP sequence 10 residues long in which two strains have a single difference, the distance will be 0.1. The clustering library uses this function to compare the strains and produce a JSON tree, which can then be rendered in the browser, using D3 (http://d3js.org) to draw a dendrogram. The dendrogram will update to reflect changes in selected strains and features.

**Figure 3.**
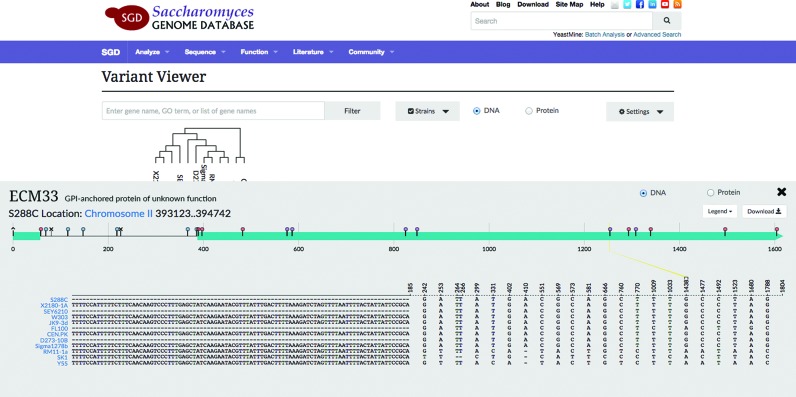
ORF view. The lollipop variant visualization highlights variation within a single ORF. In this example the ECM33 ORF nucleotide variation between 11 strain sequences is shown. SNPs, insertions and deletions are represented as symbols called lollipops. Below the lollipop visualization, the varied segments of the aligned sequences are shown. Mousing over a lollipop in the upper portion of the display highlights the corresponding sequence across the visible strains. In this view, users can also toggle between DNA and protein modes.

Clicking on a row of the matrix generates an ORF view, which provides detailed information about variants in the selected feature (Figure 3). At the top of this section of the interface is an abstract representation of the feature, with SNPs represented as small circles on top of stems, referred to as ‘lollipops.’ The design of this display was inspired by a python lollipop diagram generator (https://github.com/pbnjay/lollipops). Like the matrix, the ORF view can be toggled between DNA and protein modes. In DNA mode, the sequence visualization includes introns. In protein mode, domains are rendered along the bottom of the display, from the same data used to represent domains on the SGD protein pages (e.g. http://yeastgenome.org/locus/rad54/protein). Here, the colors for the protein domains signify the source from which SGD obtained the data. The abstract visualization of all the variants in the selected feature is visually associated with the individual changes in the sequences.

Below the lollipop variant visualization, multi-aligned sequences from the selected strains are rendered on top of each other, with the reference strain on top. The distinguishing feature of this sequence representation is the manner in which identical segments of the aligned sequences are hidden from view. The only visible characters are the ones for which there is a differing residue in the corresponding sequence position of at least one strain. With most alignment representations, identical portions of the sequence consume the most space, complicating the interface and making it difficult to see sequence variants. In contrast, this visualization emphasizes varied segments. Mousing over a variant lollipop highlights that section in the sequence display. Likewise, mousing over a sequence portion highlights the lollipop in the top portion of the display. Sometimes, the visible portions of the sequences are wider than the screen display. In this case, the user can scroll to the right to view the rest of the sequences.

## OPEN-SOURCE TECHNOLOGY

The Variant Viewer project makes use of several open-source libraries. D3 is used to assist in rendering complex visualizations. For the application's overall framework and organization, Facebook's React.js library was used (http://facebook.github.io/react). Elasticsearch is used to store, search and retrieve processed alignment data.

The code behind the Variant Viewer is freely available on GitHub. While all of SGD's source code is available at https://github.com/yeastgenome/SGDFrontend, the ORF view of the Variant Viewer has been separately componentized and documented for ease of use by others. Anyone can visualize a set of sequences and corresponding variants by formatting their data in the specified manner and then integrating with the library described in the documentation.

To encourage programmatic use, the Variant Viewer component has been registered with BioJS (7), an open-source registry for JavaScript components applicable to biology. For the Variant Viewer, as well as every registered component, the BioJS registry shows a live example with example integration code.

Availability: http://biojs.io/d/sgd_visualization.

## FUTURE DIRECTION

Future versions of this tool will improve functionality by including additional data. For example, an area of high interest is variants within intergenic regions because it is becoming increasingly clear that much phenotypic and functional variation between strains is due to differences in regulation, as opposed to differences in protein sequence. It will also be helpful to be able to ‘shift the reference,’ or switch the reference genome so that variants are called relative to the genome that is most informative for a specific area of study. This would also facilitate adding ORFs that are not present in the current reference genome, S288C. Further, we would like to represent associations between variants and phenotypes so that the interface could highlight variants with known phenotypic significance.
